# *In Vitro* Model to Evaluate the Development of Discolorations on Human Enamel Caused by Treatment with Mouth Rinses and Black Tea Considering Brushing

**DOI:** 10.1055/s-0043-1777047

**Published:** 2024-01-23

**Authors:** Sandra Sarembe, Nicole Michler, Carolin Ufer, Andreas Kiesow

**Affiliations:** 1 Fraunhofer Institute for Microstructure of Materials and Systems IMWS, Halle (Saale), Germany

**Keywords:** octenidine, chlorhexidine, benzydamine, polyhexamethylene biguanide, hexetidine, staining, black tea, tooth color

## Abstract

**Objectives** The study aimed to develop and test an *in vitro* model to investigate the staining potential of mouth rinses on human enamel, considering alternating intake of black tea and tooth brushing, thus mimicking the situation in the oral cavity more realistically.

**Materials and Methods** Eight mouth rinses with six different active ingredients (benzydamine hydrochloride [BNZ], polyhexamethylene biguanide hydrochloride [PHMB], chlorhexidine digluconate [CHX], hexetidine gluconate [HEX], octenidine dihydrochloride [OCT] and octenidine dihydrochloride + 2-phenoxyethanol [OCTP]) and concentrations were tested. Sets of six halved human molar crowns were initially pretreated by soaking in artificial saliva (30 min). Afterward, the cyclic treatment was started by soaking in artificial saliva (2 min), staining with black tea (1 min), brushing with toothpaste (5 s), and soaking in the mouth rinse (30 s). Samples were rinsed with distilled water after each treatment step. The cyclic treatment was repeated 30 times, mimicking the consumer behavior after 15 days. Photographic images were taken after 0, 10, 20, and 30 cycles. Color measurements were conducted after each staining and brushing step using a VITA-Easyshade spectrophotometer to determine the difference in lightness ∆L and the total color difference ∆E.

**Statistical Analysis** Analysis of variance and post-hoc Tukey test (α = 0.05) were applied.

**Results** The new testing model with included brushing sequences allowed to assess the staining behavior on human teeth and provided a clear differentiation between the different investigated products. In detail, up to cycle 10, ΔE values increased for all mouth rinses with each additional cycle number. However, while ΔE values continued to increase for 0.15% BNZ, 0.1% PHMB, and 0.2% CHX between treatment cycle 10 and 30, ΔE values only slightly increased after treatment with 0.08% OCTP, 0.1% OCTP, 0.1% OCT, and 0.1% HEX. After 20 and 30 cycles, significantly less staining was found for 0.08% OCTP, 0.1% OCT, 0.1% HEX as compared to 0.2% CHX, 0.15% BNZ, and 0.1% PHMB ( *p* < 0.05). ΔE-values were significantly lower after treatment with 0.1% OCTP as compared to 0.2% CHX1 and 0.2% CHX2 (p < 0.05).

**Conclusion** The proposed new methodology was found to be appropriate for assessing the staining progression of mouth rinses over a simulated application period of 15 days. The model allows differentiation of products with different active ingredients and concentrations.

## Introduction

Antiseptic mouth rinses are used in home oral care to support oral biofilm management if mechanical plaque control is temporarily impaired, for example, after oral surgical interventions, during orthodontic therapy with fixed appliances or as an additional measure for the treatment of gingivitis or periodontitis. 1 In general, antiseptic agents need to have a broad, nonspecific antibacterial efficacy toward all bacterial species as well as fungi on the one hand and a low systemic cytotoxicity on the other hand. Furthermore, a long-lasting substantivity is needed to prevent immediate washout of the active substances from the mouth by the salivary flow. 2

Currently, chlorhexidine digluconate (CHX), a bisbiguanide, is the most effective antimicrobial agent used in antiseptic mouth rinses, which has been demonstrated by a multitude of studies. 3 4 5 The cationic CHX molecules bind to the negatively charged sites of the bacterial cell membrane and to the tissue surfaces of the oral cavity, thus providing a sustained depot effect. The interaction with bacterial cells results in autolysis and cell death. However, the use of CHX containing antiseptic oral rinses can cause mucosal irritation and dysgeusia. 6 Studies have shown that CHX application promotes calculus formation. 7 Furthermore, rinsing with CHX in combination with staining dietary components can lead to accumulation of staining products on the tooth and mucosal surfaces, resulting in undesired extrinsic tooth discoloration. 8 These possible side effects of CHX led to the development of equivalent alternatives.

Alternatives like octenidine dihydrochloride (OCT) and octenidine dihydrochloride + 2-phenoxyethanol (OCTP) show better biocompatibility than CHX, 9 possibly attributable to the lack of an amide and ester structure in its molecule. 10 Besides, benzydamine hydrochloride (BNZ), polyhexamethylene biguanide hydrochloride (PHMB), and hexetidine gluconate (HEX) are further alternative antibacterial agents in oral rinsing products. 11 12 While the staining potential of CHX-containing rinsing solutions has been investigated extensively *in vitro* , there is a lack of data from products containing OCT, OCTP, BNZ, PHMB, and HEX.

The staining potential of mouth rinses is assessed either *in vivo* or *in vitro* . 13 14 In *in vivo* studies, the discoloration index provides an opportunity to estimate the extent of discoloration per tooth in the oral cavity and is often used to assess the color, intensity, and distribution of extrinsic stains among vestibular and lingual tooth surfaces. 14 Using *in vitro* models, artificial materials are often treated with saliva and mouth rinses followed by rinsing with a staining media (e.g., black tea, red wine, coffee). Color changes are assessed by recording the optical density using a ultraviolet/visible double beam spectrophotometer 15 or L*a*b values using a spectrophotometer calculating the color difference ΔE. 16 17 18 Here, the staining behavior of mouth rinses can be evaluated in comprehensive screenings over few days under standardized conditions. Preceding the *in vivo* tests, results of such screenings can be used for a preselection of the best suited rinsing components or products to be included in the investigation. Thus, the efficiency of the clinical investigations can be significantly increased considering the long time needed for *in vivo* tests (e.g., 4 weeks–6 months) for each product. 19

To provide the most useful results *in vitro* models should mirror the development of discolorations caused by application of mouth rinses including cleaning in the oral cavity as realistically as possible. On the other hand, daily cleaning with toothpaste and toothbrush is currently not considered in the existing *in vitro* models. Avoidance of integrating tooth brushing sequences in staining testing models is probably due to the detrimental effects of cleaning compared to the building up of staining, in so far, to integrate intermediate cleaning cycles in the testing protocol may result in a decrease of sensitivity and selectivity when the staining potential of oral care products is to be evaluated. Possibly, the findings of any clear differences comparing alternative products are interfered, thus impede the correlation with *in vivo* testing. At least, it may drastically increase testing cycle numbers and time to reach significance in the evaluation results, therefore essentially reducing the efficiency of *in vitro* testing.

The aim of this study was to develop and test an *in vitro* test model for investigations of the staining potential of mouth rinses on human tooth surfaces that also takes tooth brushing into account. In addition to better mimicking the reality in an oral cavity, the model provides also potential to investigate the opposite effects of discoloration-built ups by mouth rinse application in combination with dietary factors on the one hand, and tooth brushing on the other hand. This means that also the adherence of the staining to the tooth surface and its resistance against cleaning can be considered. The null hypothesis was that the staining potential of mouth rinses with different ingredients and concentrations cannot be clearly differentiated using the developed *in vitro* model.

## Materials and Methods

### Sample Preparation

Human teeth were acquired from Indiana University School of Dentistry, Oral Health Research Institute. Entire human molars with cervical dentine area were used. The roots were separated, and the crowns were sectioned in halves by a water-cooled diamond saw (Minitom, Struers). The halved tooth crowns were fixed (UHU plus endfest 300, two-component epoxy resin adhesive, UHU GmbH & Co. KG, Bühl, Germany) to a microscopic slide.

### Treatment Groups

Table 1 shows the treatment groups (mouth rinses) as well as the including actives and concentrations, the brand and manufacturer of each.

**Table 1:** Treatment products (mouth rinses)

Abbreviation of the treatment groups	Actives and concentration	Brand and manufacturer
0.15% BNZ	0.15% benzydamine hydrochloride	Tantum Verde, Angelini Pharma Deutschland GmbH, Munich, Germany
0.1% PHMB	0.1% polyhexamethylene biguanide hydrochloride	ProntOral, B. Braun SE, Melsungen, Germany
0.2% CHX1	0.2% chlorhexidine digluconate	Meridol med CHX 0.2%, CP GABA GmbH, Hamburg, Germany
0.2% CHX2	0.2% chlorhexidine digluconate	Chlorhexamed Forte, 0.2% CHX, GlaxoSmithKline Consumer Healthcare GmbH & Co. KG, Munich, Germany
0.1% HEX	0.1% hexetidine gluconate	Hexoral, Johnson & Johnson GmbH, Neuss, Germany
0.08% OCTP	Octenidine dihydrochloride, < 1% 2-phenoxyethanol	Octenident, Schülke & Mayr GmbH, Norderstedt, Germany
0.1% OCTP	0.1% octenidine dihydrochloride, 2% 2-phenoxyethanol	Octenisept, Schülke & Mayr GmbH, Norderstedt, Germany
0.1% OCT	0.1% octenidine dihydrochloride	Octenident antiseptic, Schülke & Mayr GmbH, Norderstedt, Germany

### Experimental Approach

**Fig. 1:**
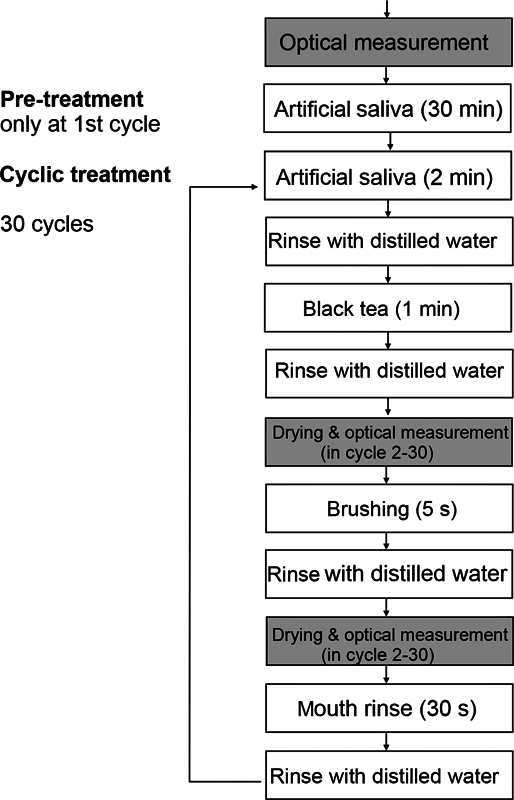
Developed test procedure.

The test procedure is depicted in Fig. 1 . The experiments were designed to simulate a 15-day consumer application period, assuming two applications of the rinsing solution per day (30 applications in total) and tooth brushing twice daily (30 applications in total). Sets of six specimens were placed in a 50 mL centrifuge tube. The specimens were pretreated with artificial saliva 20 for 30 minutes at 37°C to simulate the pellicle formation. Afterward, the specimens were shortly rinsed in a beaker with 200 mL of distilled water under standardized conditions. For the subsequent cyclic procedure, specimens were stored in artificial saliva (2 min), rinsed with distilled water, exposed to warm black tea (50°C, 1 min) and rinsed with distilled water again, followed by an optical measurement (only in cycle 2–30). Then the specimens were brushed for 5 seconds, rinsed with distilled water, and measured again optically (only in cycle 2–30). Afterward, the specimens were placed in rinsing solution (30 s) and rinsed with distilled water. The total number of cycles was 30. Ten cycles were performed per day on 3 consecutive days. Samples were dried before each optical measurement.

A standard tea solution was made using Typhoo One cup (Typhoo, United Kingdom). To receive a defined concentration, the solution was prepared by brewing one bag of tea in 100 mL of freshly boiled distilled water for 5 minutes. After stirring and filtering through double layer gauze, the tea solution was stored for distribution at 50°C. Black tea and rinsing solution were renewed after every second cycle.

Before brushing, the toothbrushes (Oral-B Indicator 35 medium, Procter & Gamble Company, Cincinnati, Ohio, United States) were wetted with distilled water under standardized conditions. Brushing was performed in a brushing simulator (ZM 3-8, SD-Mechatronik, Feldkirchen-Westerham, Germany) with zig-zag movements and an applied load of 2 N (stroke frequency 1.6 Hz). A time of 5 seconds for brushing a tooth is assumed to be representative; therefore, 5 seconds per tooth sample per cycle were chosen. 21 The toothpaste was applied as an aqueous slurry. Therefore, the toothpaste (medium abrasive Colgate Total, Colgate Palmolive Company, New York City, United States) was used at a dilution of 1:3 with water (w/w). The samples were carefully rinsed with distilled water after brushing treatment.

### Photographs and Color Measurements

Photographic images were taken before the start, after 10, 20, and 30 cycles with a reflex camera (Canon EOS 600D, Canon GmbH, Munich, Germany) using a photo measuring point, a black sample background, and with always the same camera settings to demonstrate possible staining effects. Color measurements were conducted after each staining and brushing step, that is, 20 times per day using a VITA Easyshade Compact spectrophotometer (VITA Zahnfabrik, Bad Säckingen, Germany).

All color measurements were performed in the same room and from the same operator. In accordance with the manufacturer's instructions, the spectrophotometer was calibrated by measuring a white disc before taking measurements. After each human molar was placed on a neutral background, the tip of the spectrophotometer was placed in contact with and perpendicular to the middle of the tooth.

Values were recorded using the Hunter L*a*b* color scale, which is a color space with coordinates for lightness (white-black, L*), where the maximum for L* is 100 (a perfect reflecting diffuser) and the minimum would be zero (black). The scale also represents redness-greenness (a*), where negative a* is green and positive a* is red, and yellowness-blueness (b*), where negative b* is blue and positive b* is yellow.

ΔE, which is the total color difference between initial situation (before staining) and treated specimens (after staining or staining and brushing), is calculated according to the following equation:



The normality of the mean ΔE values was analyzed using the Shapiro–Wilk test. The normally distributed data was statistical analyzed using one-way analysis of variance with post-hoc Tukey honestly significant difference-test and Levene's test for analyses of homogeneity of variance (Origin2019b, OriginLab Corporation Company, United States). The level of significance α was set to 0.05.

## Results

**Fig. 2:**
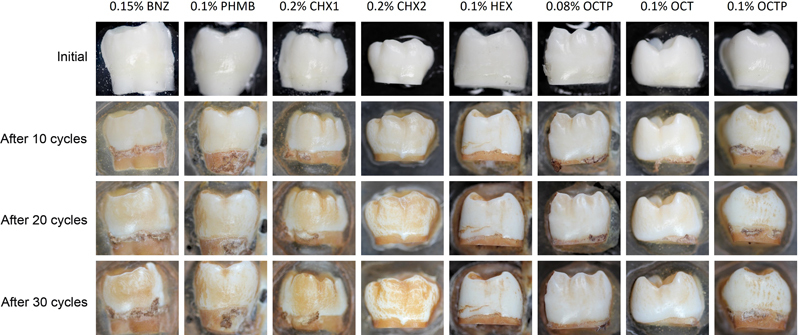
Exemplary photographic images of the enamel specimens before and after 10, 20, 30 treatment cycles with different rinsing solutions in combination with black tea. BNZ, benzydamine hydrochloride; CHX, chlorhexidine digluconate; HEX, hexetidine gluconate; OCT, octenidine dihydrochloride; OCTP, octenidine dihydrochloride + 2-phenoxyethanol; PHMB, polyhexamethylene biguanide hydrochloride.

**Fig. 3:**
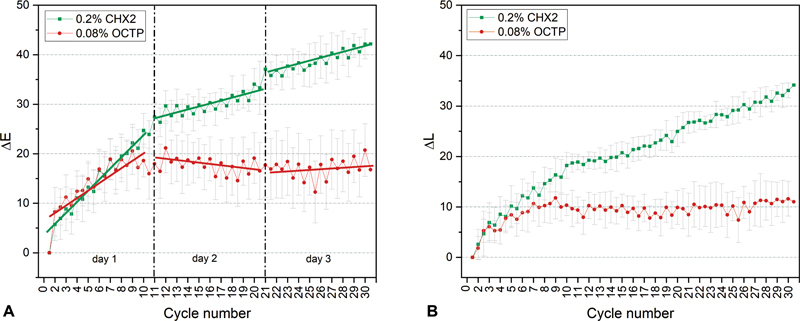
Exemplary progression of the ∆E ( **A** ) and ∆L ( **B** ) mean values (with standard deviation) of the human enamel samples during treatment with 0.2% chlorhexidine digluconate 2 (CHX2) and 0.08% octenidine dihydrochloride + 2-phenoxyethanol (OCTP).

**Fig. 4:**
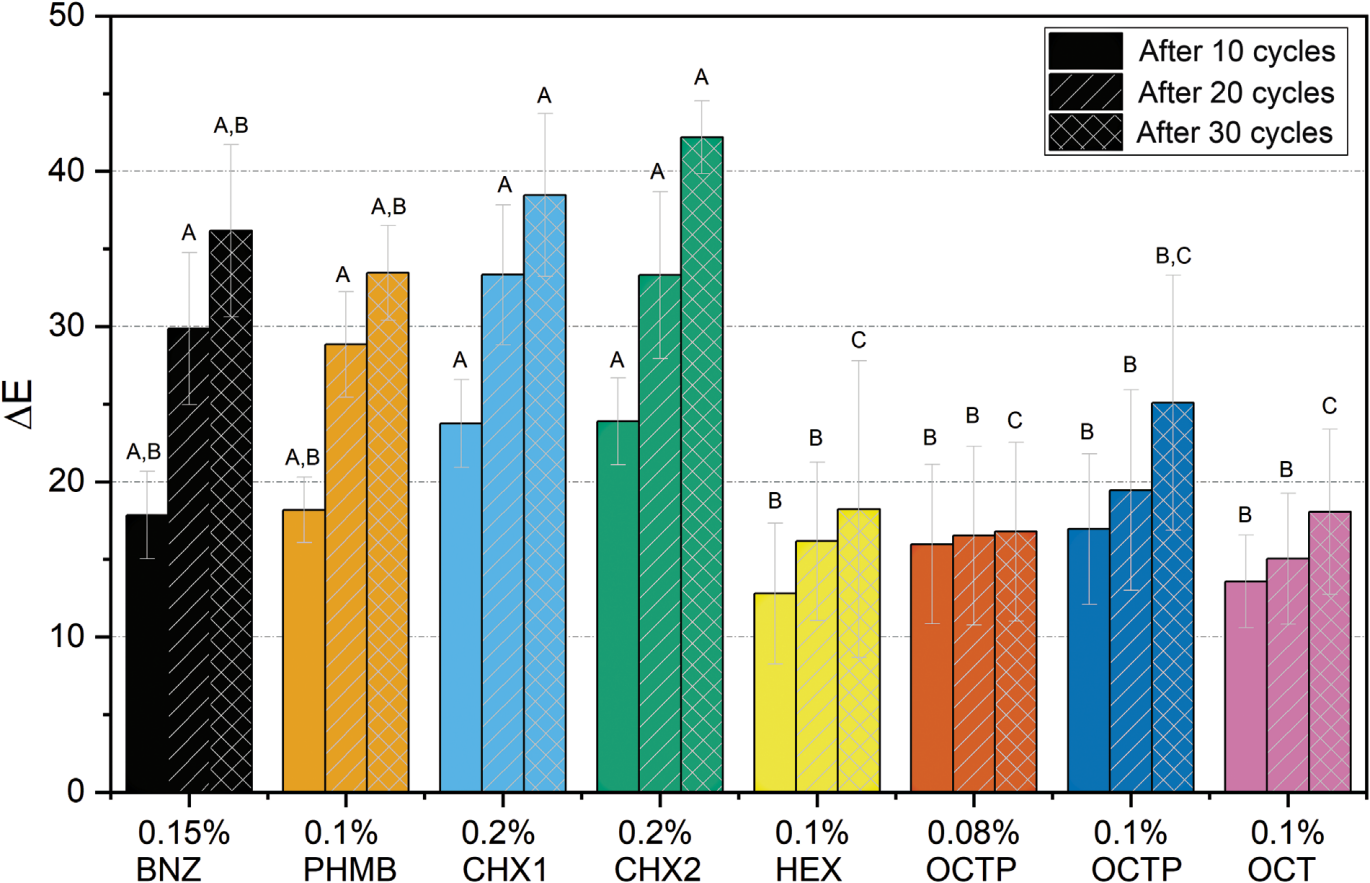
∆E mean values (with standard deviation) of the human enamel samples after treatment with different rinsing solutions: after 10 cycles, after 20 cycles, after 30 cycles; Mean values that do not have a common letter are significantly different ( *p* ≤ 0.05). BNZ, benzydamine hydrochloride; CHX, chlorhexidine digluconate; HEX, hexetidine gluconate; OCT, octenidine dihydrochloride; OCTP, octenidine dihydrochloride + 2-phenoxyethanol; PHMB, polyhexamethylene biguanide hydrochloride.

In Fig. 2 , exemplary images are shown for one enamel sample per test group, for the initial state and the state after 10, 20, and 30 treatment cycles. The respective ∆L and ∆E values determined by the color measurements are shown in Figs 3 to 4 and in the Supplementary Material Figs. 1–2 (available in the online version). The results of the statistical analysis are presented in the Supplementary Material Tables 1 to 3 (available in the online version).

Fig. 3 shows a representative progression of the ΔE and ΔL values for the products that caused the highest (0.2% CHX2) and lowest staining intensity (0.08% OCTP). ΔE and ΔL of the enamel surfaces increased after treatment with all mouth rinses. Due to the alternating staining and brushing, a zig-zag progression of the ΔE data was obtained ( Fig. 3 , Supplementary Material Fig. 1 , available in the online version). For ΔL data, the zig-zag progression is less pronounced ( Supplementary Material Fig. 2 , available in the online version). The results show that black tea staining was not completely removed by brushing with toothpaste during each cycle. Up to 10 cycles, ΔL and ΔE values increased for all mouth rinses with each additional cycle number, that is, the samples became progressively darker, and black tea staining built up continuously. Between treatment cycle 10 and 30, the ΔL and ΔE values reached a plateau after treatment with 0.08% OCTP, while ΔL and ΔE values continued to increase for the treatment with 0.2% CHX2, thus contributing to further discoloration. Similar progression curves as shown for 0.08% OCTP were also observed for 0.1% OCTP, 0.1 OCT, and 0.1% HEX ( Supplementary Material Figs. 1B and 2B , available in the online version). For 0.15% BNZ, 0.1% PHMB, 0.2% CHX1, the curve progression of ΔL and ΔE was comparable to the one of 0.2% CHX2 ( Supplementary Material Figs. 1A and 2B , available in the online version).

Fig. 4 shows the mean ΔE values of the human enamel samples after 10, 20, and 30 cycles. After cycle 10, lowest staining was measured for treatment with 0.1% HEX and 0.1% OCT followed by 0.08% OCTP and 0.1% OCTP. Significant differences were found for 0.1% HEX, 0.08% OCTP, 0.1% OCTP, and 0.1% OCT as compared to 0.2% CHX1 and 0.2% CHX2 ( Fig. 4 ). After cycle 20, significantly lower staining was determined for, 0.1% HEX, 0.08% OCTP, 0.1% OCTP, and 0.1% OCT as compared to all other tested rinsing solutions. After cycle 30, the lowest staining was recorded for treatment with 0.08% OCTP, 0.1% HEX, and 0.1% OCT followed by 0.1% OCTP. Significant differences were determined for 0.1% HEX, 0.08% OCTP, and 0.1% OCT as compared to 0.15% BNZ, 0.1% PHMB, 0.2% CHX1, and 0.2% CHX2. Furthermore, ΔE-values were significantly lower after treatment with 0.1% OCTP as compared to 0.2% CHX1 and 0.2% CHX2.

## Discussion

The experimental approach of this study is new and partially exploratory in nature. It shows changes in the tooth color after soaking in antiseptic oral rinsing solutions and black tea as well as after tooth brushing over a simulated application period of 15 days. To the authors' knowledge, no *in vitro* data on the development of discoloration on human teeth after cyclic staining treatment including toothbrushing have been published so far. Most of the published studies only investigate the staining potential of mouth rinses in combination with coloring foods 18 22 or examine the ability of oral care products (e.g., toothpastes) to remove tooth staining. 17 23

To investigate the tooth color changes *in vitro* , samples are usually treated cyclically 16 24 or continuously. 25 26 To assess the tooth color, different instrumental objective measurements using spectrophotometers, colorimeters, and image analysis techniques are performed. 8 First, Prayitno and Addy developed an *in vitro* model to assess dental discolorations caused by rinsing with CHX solutions of different concentrations in combination with black tea. 15 Therefore, transparent polymethyl methacrylate (PMMA) specimens were alternately stained by exposing to saliva, a CHX-containing mouth rinse and a black tea solution. PMMA is used due to a high degree of standardization. A study has shown that these *in vitro* tests (without any consideration of daily mechanical cleaning) are in qualitatively agreement with the clinical situation. 13 Discoloration appears by interaction between the positively charged active (CHX) and the anionic staining dietary components. The deposited complexes produced *in vitro* are identical in color to those seen on teeth clinically. 27 CHX alone does not stain but binds selectively to the anionic staining dietary chromogens, causing discoloration. 24 It is also stated that a salivary pellicle is not essential to perform discoloration but acts to enhance staining. 27

The experimental approach 15 which is characterized by cyclic rinsing of PMMA samples with saliva, antiseptic mouth rinses and black tea, was also used with an integrated brushing step to assess the stain removal by toothbrushes and toothpastes. 28 29 However, since the same study performed *in vivo* and *in vitro* showed different results, the author's concluded that the findings of the *in vitro* approach are unlikely to be of clinical significance and models need to be developed on dental hard tissues. 28 30 Therefore, human tooth crowns were used in this study instead of artificial materials to develop an *in vitro* model that mimics the oral situation more accurately and may better predict the progression of discoloration caused by antiseptic mouth rinses and black tea considering tooth brushing.

In this *in vitro* model, treatment times with mouth rinses, staining with black tea, and tooth brushing were adapted to simulate the situation in the oral cavity in real life as close as possible. The treatments were highly standardized and controlled. Extremely long treatment times in the respective medium, as also stated in literature, 26 31 were omitted. In addition, the tooth color was measured after each staining cycle as well as after each brushing step to monitor the progressive stain development, rather than only once at the end of the experimental procedure.

Brushing with toothpaste was included to mirror oral hygiene practices as per general recommendations. It means that also the adherence of the staining products to the tooth surface and their resistance against cleaning is considered in the test results. It is also reflected in the measurement results that the brushing step is effective in reducing stain, although complete stain removal was not achieved. The applied discoloration is thus gradually accumulated with increasing number of cycles, allowing also to assess the staining kinetics as a function of cycle numbers.

The null hypothesis of the study has to be rejected (see Fig. 4 ) since these findings are in contrast to the assumption that including the detrimental effects of intermediate tooth cleaning in a staining testing model will impede the sensitivity and significance of the results or drastically reduce the effectiveness of the *in vitro* testing. It was shown that the proposed model allowed to quantify both the discoloration intensity and kinetics for all products investigated. In particular, the results allowed a clear differentiation between different mouth rinses, demonstrating the applicability of the model for a more realistic *in vitro* evaluation of the staining potential of oral care products.

Regarding a detailed interpretation of the observed differences for the investigated products, it has to be considered that little background information on the composition of the formulations was available. Therefore, it is difficult to derive further conclusions from these findings on the actual active interaction and the dependence on concentration. The high staining of CHX in combination with black tea was expected (as already mentioned) and can be explained by binding of positively charged active ingredient on all negatively charged dental surfaces. 8 Since all other tested mouth rinses include cationic active ingredients (BNZ, PHMB, HEX, OCT, OCTP), the same or similar staining mechanisms can be assumed. The observed differences in staining over time may be attributed to different degrees of adhesion of the active ingredient to the pellicle or enamel surface. It is conceivable that the accumulated staining caused by treatment with OCT, OCTP and HEX can be more easily removed by brushing, as demonstrated by only a slight increase in staining over time.

A less pronounced staining of teeth and dentures by HEX has also been described as compared to CHX 12 32 and this study results confirm earlier findings from *in vivo* studies reported in literature. Furthermore, mouth rinses with active ingredients BNZ and PHMB caused a higher dental staining as compared to OCT and OCTP and lower staining than CHX in this study. The results of this study are also in good agreement with the literature, 11 where BNZ was found to cause lower light and dark shades on a provisional acrylic resin than after treatment with a CHX-containing mouth rinse. These agreements underline the applicability of the new model. For PHMB, no data in context of dental discolorations were found in the literature.

However, the staining potential of antiseptic mouth rinses is an undesirable side effect compared to the products efficacy—the antibacterial effect. However, some authors describe that the degree of dental staining caused by CHX-containing mouth rinses can also be used as an indicator for the antimicrobial activity. 33 Nevertheless, it is currently not known whether this statement also applies to other antiseptic agents (e.g., for OCT). In comparison to CHX, OCT showed a similar or even stronger antibacterial effect in clinical studies 34 35 and according to a recent review OCT is considered either superior or comparable to CHX-based mouthwashes in controlling dental plaque. 36 The staining potential can be described as mild 37 or as generally removable by toothbrushing. 38 39 40 As shown in the literature, relatively little formulations have been studied in this regard and further studies focusing on antimicrobial activity in relation to staining potential should be conducted.

The developed model could be a beneficial tool due the consideration of more realistic treatment conditions and the sensitivity and selectivity regarding different rinsing components as demonstrated in this study.

## Conclusion

A new testing model for the *in vivo* assessment of the staining potential of oral care mouth rinses has been proposed and successfully tested. The testing procedure is based on staining treatments of human teeth but includes also tooth brushing cycles to mimic the reality of dental hygiene and to consider the adherence of staining products to the tooth surface. It was shown that the testing model allows a clear differentiation of different mouth rinses regarding staining intensity and kinetics.
