# Supplementary material for: In Vitro Model to Evaluate the Development of Discolorations on Human Enamel Caused by Treatment with Mouth Rinses and Black Tea Considering Brushing
Source: Eur J Dent. 2024 Jan 23;18(3):925–32. doi: 10.1055/s-0043-1777047 (PMC11290939; doi:10.1055/s-0043-1777047)
Supplement: Supplementary file 1 — Supplementary Material [file 10-1055-s-0043-1777047-s2382923.pdf]

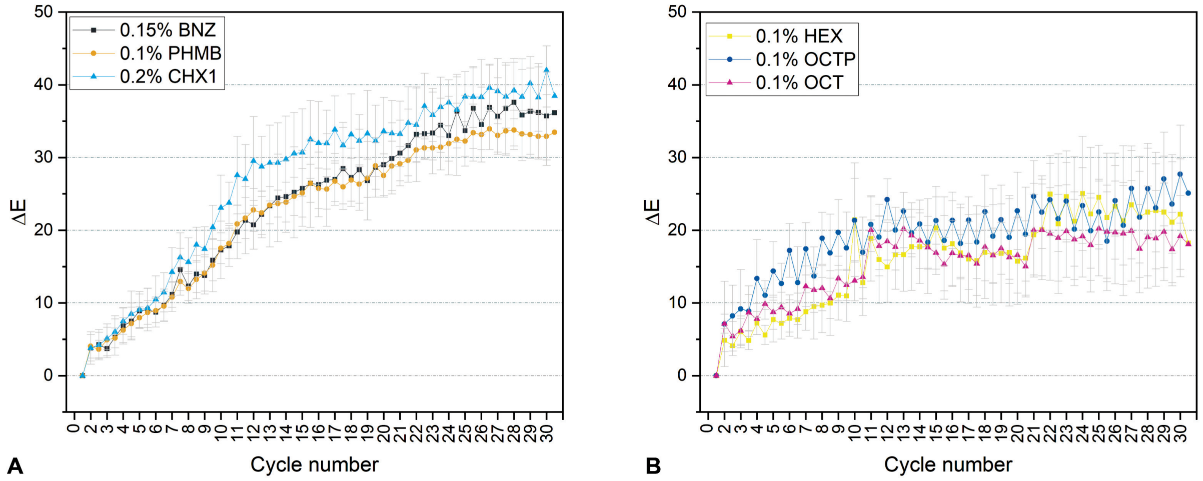

Supplementary Material Fig. 1 Exemplary progression of the ΔE mean values (with standard deviation) of the human enamel samples during treatment: (**A**) with 0.15% benzydamine hydrochloride (BNZ), 0.1% polyhexamethylene biguanide hydrochloride (PHMB), 0.1% chlorhexidine digluconate (CHX1), (**B**) with 0.1% hexetidine gluconate (HEX), 0.1% octenidine dihydrochloride + 2-phenoxyethanol (OCTP), 0.1% octenidine dihydrochloride (OCT).

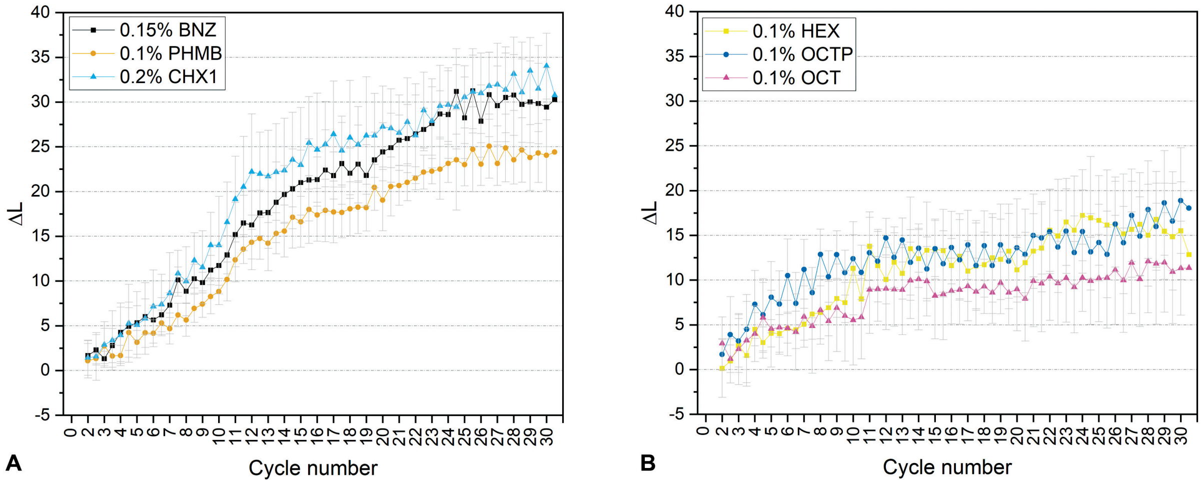

Supplementary Material Fig. 2 Exemplary progression of the ΔL mean values (with standard deviation) of the human enamel samples during treatment: (**A**) with 0.15% benzydamine hydrochloride (BNZ), 0.1% polyhexamethylene biguanide hydrochloride (PHMB), 0.1% chlorhexidine digluconate (CHX1), (**B**) with 0.1% hexetidine gluconate (HEX), 0.1% octenidine dihydrochloride + 2-phenoxyethanol (OCTP), 0.1% octenidine dihydrochloride (OCT).

 © 2024. The Author(s).

Supplementary Material Table 1 Results of the statistics (Tukey test) regarding the color change ΔE after 10 cycles between treatment products

	p-Value		*p*-Value
0.1% PHMB vs. 0.15%BNZ	1	0.08% OCTP vs. 0.1% HEX	0.79
0.2% CHX1 vs. 0.15% BNZ	0.12	0.1% OCTP vs. 0.15% BNZ	1
0.2% CHX1 vs. 0.1% PHMB	0.17	0.1% OCTP vs. 0.1% PHMB	1
0.2% CHX2 vs. 0.15% BNZ	0.11	0.1% OCTP vs. 0.2% CHX1	0.05[a]
0.2%CHX2 vs. 0.1% PHMB	0.15	0.1% OCTP vs. 0.2% CHX2	0.04[a]
0.2% CHX2 vs. 0.2% CHX1	1	0.1% OCTP vs. 0.1% HEX	0.51
0.1% HEX vs. 0.15% BNZ	0.27	0.1% OCTP vs. 0.1% OCTP	1
0.1% HEX vs. 0.1% PHMB	0.20	0.1% OCT vs. 0.15% BNZ	0.48
0.1% HEX vs. 0.2% CHX1	<0.001[a]	0.1% OCT vs. 0.1% PHMB	0.38
0.1% HEX vs. 0.2% CHX2	<0.001[a]	0.1% OCT vs. 0.2% CHX1	<0.001[a]
0.08% OCTP vs. 0.15% BNZ	0.99	0.1% OCT vs. 0.2% CHX2	<0.001[a]
0.08% OCTP vs. 0.1% PHMB	0.96	0.1% OCT vs. 0.1% HEX	1
0.08% OCTP vs. 0.2% CHX1	0.01[a]	0.1% OCT vs. 0.1% OCTP	0.94
0.08% OCTP vs. 0.2% CHX2	0.01[a]	0.1% OCT vs. 0.08% OCTP	0.75

Abbreviations: BNZ, benzydamine hydrochloride; CHX, chlorhexidine digluconate; HEX, hexetidine gluconate; OCT, octenidine dihydrochloride; OCTP, octenidine dihydrochloride + 2-phenoxyethanol; PHMB, polyhexamethylene biguanide hydrochloride.
p-Values; [a]significant differences <0.05.

Supplementary Material Table 2 Results of the statistics (Tukey test) regarding the color change ΔE after 20 cycles between treatment products

	p-Value		*p*-Value
0.1% PHMB vs. 0.15%BNZ	1	0.08% OCTP vs. 0.1% HEX	1
0.2% CHX1 vs. 0.15% BNZ	0.93	0.1% OCTP vs. 0.15% BNZ	0.02[a]
0.2% CHX1 vs. 0.1% PHMB	0.78	0.1% OCTP vs. 0.1% PHMB	0.05[a]
0.2% CHX2 vs. 0.15% BNZ	0.93	0.1% OCTP vs. 0.2% CHX1	<0.001[a]
0.2%CHX2 vs. 0.1% PHMB	0.78	0.1% OCTP vs. 0.2% CHX2	<0.001[a]
0.2% CHX2 vs. 0.2% CHX1	1	0.1% OCTP vs. 0.1% HEX	0.94
0.1% HEX vs. 0.15% BNZ	<0.001[a]	0.1% OCTP vs. 0.1% OCTP	0.97
0.1% HEX vs. 0.1% PHMB	0.002[a]	0.1% OCT vs. 0.15% BNZ	<0.001[a]
0.1% HEX vs. 0.2% CHX1	<0.001[a]	0.1% OCT vs. 0.1% PHMB	<0.001[a]
0.1% HEX vs. 0.2% CHX2	<0.001[a]	0.1% OCT vs. 0.2% CHX1	<0.001[a]
0.08% OCTP vs. 0.15% BNZ	0.001[a]	0.1% OCT vs. 0.2% CHX2	<0.001[a]
0.08% OCTP vs. 0.1% PHMB	0.003	0.1% OCT vs. 0.1% HEX	1
0.08% OCTP vs. 0.2% CHX1	<0.001[a]	0.1% OCT vs. 0.1% OCTP	1
0.08% OCTP vs. 0.2% CHX2	<0.001[a]	0.1% OCT vs. 0.08% OCTP	0.79

Abbreviations: BNZ, benzydamine hydrochloride; CHX, chlorhexidine digluconate; HEX, hexetidine gluconate; OCT, octenidine dihydrochloride; OCTP, octenidine dihydrochloride + 2-phenoxyethanol; PHMB, polyhexamethylene biguanide hydrochloride.
p-Values; [a]significant differences <0.05.

 © 2024. The Author(s).

Supplementary Material Table 3 Results of the statistics (Tukey test) regarding the color change ΔE after 30 cycles between treatment products

	p-Value		*p*-Value
0.1% PHMB vs. 0.15%BNZ	0.99	0.08% OCTP vs. 0.1% HEX	1
0.2% CHX1 vs. 0.15% BNZ	1	0.1% OCTP vs. 0.15% BNZ	0.05
0.2% CHX1 vs. 0.1% PHMB	0.84	0.1% OCTP vs. 0.1% PHMB	0.27
0.2% CHX2 vs. 0.15% BNZ	0.67	0.1% OCTP vs. 0.2% CHX1	0.01[a]
0.2%CHX2 vs. 0.1% PHMB	0.22	0.1% OCTP vs. 0.2% CHX2	<0.001[a]
0.2% CHX2 vs. 0.2% CHX1	0.96	0.1% OCTP vs. 0.1% HEX	0.52
0.1% HEX vs. 0.15% BNZ	<0.001[a]	0.1% OCTP vs. 0.1% OCTP	0.28
0.1% HEX vs. 0.1% PHMB	0.002[a]	0.1% OCT vs. 0.15% BNZ	<0.001[a]
0.1% HEX vs. 0.2% CHX1	<0.001[a]	0.1% OCT vs. 0.1% PHMB	0.002[a]
0.1% HEX vs. 0.2% CHX2	<0.001[a]	0.1% OCT vs. 0.2% CHX1	<0.001[a]
0.08% OCTP vs. 0.15% BNZ	<0.001[a]	0.1% OCT vs. 0.2% CHX2	<0.001[a]
0.08% OCTP vs. 0.1% PHMB	<0.001[a]	0.1% OCT vs. 0.1% HEX	1
0.08% OCTP vs. 0.2% CHX1	<0.001[a]	0.1% OCT vs. 0.1% OCTP	1
0.08% OCTP vs. 0.2% CHX2	<0.001[a]	0.1% OCT vs. 0.08% OCTP	0.49

Abbreviations: BNZ, benzydamine hydrochloride; CHX, chlorhexidine digluconate; HEX, hexetidine gluconate; OCT, octenidine dihydrochloride; OCTP, octenidine dihydrochloride + 2-phenoxyethanol; PHMB, polyhexamethylene biguanide hydrochloride.
p-Values; [a]significant differences <0.05.

 © 2024. The Author(s).
